# Cultivation of Keratinocytes and Fibroblasts in a Three-Dimensional Bovine Collagen-Elastin Matrix (Matriderm^®^) and Application for Full Thickness Wound Coverage *in Vivo*

**DOI:** 10.3390/ijms140714460

**Published:** 2013-07-11

**Authors:** Jasper Killat, Kerstin Reimers, Claudia Y. Choi, Sabrina Jahn, Peter M. Vogt, Christine Radtke

**Affiliations:** Department of Plastic, Hand- and Reconstructive Surgery, Hannover Medical School, Hannover D-30659, Germany; E-Mails: jasper.killat@gmail.com (J.K.); reimers.kerstin@mh-hannover.de (K.R.); claudia.choi@klinikum-bremen-mitte.de (C.Y.C.) jahn.sabrina@mh-hannover.de (S.J.); vogt.peter@mh-hannover.de (P.M.V.)

**Keywords:** skin substitute, elastin-collagen bovine matrix, Matriderm^®^, epidermal stem cells, fibroblasts, keratinocytes

## Abstract

New skin substitutes for burn medicine or reconstructive surgery pose an important issue in plastic surgery. Matriderm^®^ is a clinically approved three-dimensional bovine collagen-elastin matrix which is already used as a dermal substitute of full thickness burn wounds. The drawback of an avital matrix is the limited integration in full thickness skin defects, depending on the defect size. To further optimize this process, Matriderm^®^ has also been studied as a matrix for tissue engineering of skin albeit long-term cultivation of the matrix with cells has been difficult. Cells have generally been seeded onto the matrix with high cell loss and minimal time-consuming migration. Here we developed a cell seeded skin equivalent after microtransfer of cells directly into the matrix. First, cells were cultured, and microinjected into Matriderm^®^. Then, cell viability in the matrix was determined by histology *in vitro*. As a next step, the skin substitute was applied *in vivo* into a full thickness rodent wound model. The wound coverage and healing was observed over a period of two weeks followed by histological examination assessing cell viability, proliferation and integration into the host. Viable and proliferating cells could be found throughout the entire matrix. The presented skin substitute resembles healthy skin in morphology and integrity. Based on this study, future investigations are planned to examine behaviour of epidermal stem cells injected into a collagen-elastin matrix under the aspects of establishment of stem cell niches and differentiation.

## 1. Introduction

### 1.1. Clinical Background

The human skin fulfills vitally important functions, e.g., it poses a barrier to infections, has an important role in the regulation of the body temperature and volume management. Moreover, it constitutes a mechanical, thermal and chemical barrier to infections.

Patients who suffer from burn- or other injuries with loss of a high percentage of total body surface area (TBSA) are confronted with various severe problems beginning with an impaired function of the affected area (*i.e.*, pulmonary insufficiency in (circular) thoracic burns, impaired movement in limb burns) and bacterial colonization to the so-called “burn disease”. The pathophysiology of burn injury is determined on a cellular and a macro level: the exposure of high temperature to the skin causes a local denaturation of proteins. Furthermore, the activation of mediators (*i.e.*, cytokines) leads to a local inflammation causing oxidants and proteolytic enzymes to be released. Capillary damage causes thrombosis and skin ischemia [1]. Thus the skin is not only affected by the trauma caused by heat itself but also by the following pathophysiological effects. The phases of wound healing are controlled by a complex cascade of cytokines. A prolonged inflammation and/or secondary (bacterial) infection can cause a dysregulation and impairment of wound healing [2]. Burn injuries have to be graded by depth and width. Burn injuries can also cause systemic problems from heart insufficiency to renal failure and metabolic disorders. Therefore skin substitutes which measure up to the functions of healthy skin can prevent patients from suffering from various severe complications. Skin substitution which can be performed in a one-stage procedure also reduces the number of necessary surgeries and thus the likeliness for complications.

Skin transplants are used in plastic and reconstructive surgery for the treatment of burn patients after trauma or malignant disease, in chronic wounds (*i.e.*, chronic venous- and diabetic ulcer) and in various other skin diseases (e.g., Toxic epidermal necrolysis, TEN). Common techniques include mesh grafting (*i.e.*, split thickness graft, STG), fullthickness skin transplantation (full thickness graft, FTG) and sandwich technique as a combination of both (according to Alexander *et al*. [3]). The treatment of a burn wound depends on the burn depth and the affected TBSA [4]. A study investigating treatments of U.S. soldiers with burn injuries revealed that from an involved TBSA of 30% to 40% patients received a cryopreserved allograft (CPA) in 50% of the cases. With a TBSA of >50% almost all patients received CPA [5]. Some authors argue that from a TBSA > 70% autologous transplantation is no longer possible [6]. However, in patients who undergo temporary skin substitution scarring is frequent [7]. Further problems such as insufficient elasticity, secondary infection and graft rejection are common. As an alternative, epidermal sheets produced from autologous cells might be used. In the case of cultured epithelial autografts (CEA), high costs of sheet fabrication and the necessity of a large staff are further problems ruling out their application in many cases. Therefore, alternative and improved methods of skin substitution are an important focus of research.

### 1.2. Skin Substitutes as an Alternative for Skin Transplants

Research on skin transplantation and especially skin substitutes is an important field in experimental surgery and subject to further development. The establishment of a skin substitute that resembles healthy human skin for long-term use has not been accomplished yet although various skin substitutes have been tested with regard to their epithelial regeneration, likeliness for secondary bacterial infection and aesthetical outcome supporting the use of cultured skin substitutes in foot ulcers [8]. Furthermore the application of (autologous) keratinocytes to matrices has been complicated in handling, low cell viability, demanding in staff requirements and therefore expensive and less implemented in a clinical setting [9].

Previous studies described the use of Matriderm^®^ and Integra^®^, a porous matrix of cross-linked bovine tendon collagen and glycosaminoglycan [10,11]. Also Biobrane^®^, a two layered silicone-nylon matrix [12], has been evaluated in its function and found an efficient carrier for keratinocytes [13]. There have been manifold approaches introducing new dermal substitutes, e.g., composite grafts, tissue engineered skin based on stem cells and scaffolds based on spider silk showing development of epithelial stratification [14]. Jin *et al.* evaluated the characteristics of plant extracted nanofibers as a scaffold for adipose derived stem cells (ASCs) and could induce their epidermal differentiation [15]. There have been different methods used in applying (stem) cells to wounds including spraying of the cells onto the wound improving wound healing [16,17]. The seeding of Matriderm^®^ as a matrix has so far been described with the application of cells on top of a matrix, resulting in a time-consuming process of migration of cells into the matrix [18].

The objective of this study was to establish of a clinical applicable skin substitute with three-dimensional skin cell cultivation within the matrix to obtain a possible resilient permanent skin substitute after major skin loss. The cells used in our study were two different lineages of fibroblasts and one lineage of keratinocytes. They were injected into Matriderm^®^ using a unique microtechnique specifically developed for this study. Our resulting skin substitute then was examined *in vitro* and *in vivo*. For *in vivo* analysis the seeded sheets were transplanted into a rodent wound model in mice’s dorsum. Cell migration, viability and proliferation within the matrix were determined *in vitro* and *in vivo* as well as the integration into normal skin after transplantation of the substitute in full thickness wounds *in vivo*. The performed examinations were thought to provide us with a broad understanding of culturing of Matriderm^®^, the effectiveness of the process of microinjection and the qualities of the assembled skin substitute for wound coverage.

## 2. Results and Discussion

### 2.1. Viability of Keratinocytes and Fibroblasts Transplanted into Matriderm^®^

JB6, NIH/3T3- and MEF- lineage were used for microtransplantation and three-dimensional cultivation in the described elastin-collagen matrix. The cells used in this study are commercially available established cell lines: JB6 cells are keratinocytes from murine epidermal tissue and NIH/3T3- and MEF- lineage are mouse embryonic fibroblasts. The cells are confluent after two to three days, which makes high cell counts possible in short time. In preliminary studies these cells were seeded onto samples of Matriderm^®^ resulting in a sparse population of the matrix with insufficient assembly and adherence (data not shown). In order to enhance the efficiency of the seeding, micro-transplantation technique for direct transplantation of single cell solution into the matrix was developed.

Following cell transplantation into the matrix, cell vitality was assessed using a membrane permeable substrate which is cleaved by cellular esterases to a green fluorescing product in living cells while dead cells are indicated by incorporation of a membrane impermeable intercalator resulting in red fluorescence of the nuclei. Green fluorescence was observed in all samples independent of injection modus and cell lineage. There was an agglomeration of cells at the site of injection directly after transplantation proving a sufficient injection technique. Here, it could be demonstrated that the newly developed technique of injection did not result in cell membrane rupture and cell loss. Also, the maintenance *in vitro* of the seeded sheets was sufficient and the nourishment of the cells within the matrix with culture medium by diffusion was possible.

The cell migration is essential for the substitute’s functionality and was therefore one of our interests. Cell migration from the site of transplantation into deeper and distant layers of the matrix could be observed. On Day 3, viable cells have migrated throughout the entire matrix in longitudinal and transversal direction in the 1mm Matriderm^®^ group as well as in the 2 mm Matriderm^®^ group. It was important for us to assess both thicknesses of available Matriderm^®^- 1 mm and 2 mm. The 2 mm Matriderm^®^ thickness has higher clinical importance since better wound regeneration is seen with a thicker matrix. Still the nourishment of cells in a thicker matrix is harder to achieve making the culturing of a seeded sheet harder.

**Figure 1:**
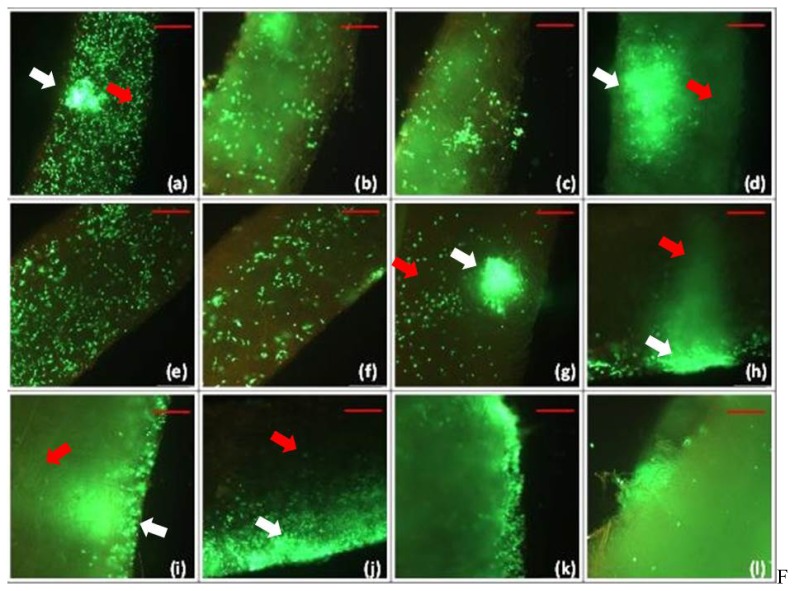
Cells of lineage JB6 in LIVE/DEAD assay. Viable cells are shown green, dead cells red. White arrows represent apical, basal layers of the matrix. Fluid milieu, 1 mm Matriderm^®^: (**a**) Day 1: At the site of injection an agglomeration of cells is visible (white arrow). The cells’ migration is proven by their distribution in longitudinal and transversal direction from the site of injection to basal layers of the matrix (red arrow); (**b**) Day 2: The Cells migrate further particularly in transversal direction; and (**c**) Day 3: Most cells are viable, accumulations of them are now found on the site opposite of injection- again proving their migration. Dry milieu, 1 mm Matriderm^®^: (**d**) Day 1: Cell agglomeration is found preliminary at the site of injection (white arrow); (**e**) Day 2: Cells migrate in longitudinal and transversal direction from site of injection; and (**f**) Day 3: Cells are found even more further from point of injection. Fluid milieu, 2 mm Matriderm^®^: (**g**) Day 1: An agglomeration of cells is found at the site of injection (white arrow); (**h**) Day 2: Cells migrate in longitudinal direction away from the site of injection; and (**i**) Day 3: Cells distribute further throughout the matrix, preliminary on the surface but also into deeper layers. Dry milieu, 2 mm Matriderm^®^: (**j**) Day 1: Accumulation of cells found at the site of injection (white arrow); (**k**) Day 2: Cells distribute further throughout the matrix; and (**l**) Day 3: Alive cells are still found. Scale bar = 500 μm.

We discriminated between two groups of pre-treatment of Matriderm^®^. In one group Matriderm^®^ was not pre-treated before being injected with keratinocytes and fibroblasts (dry milieu). In the other group Matriderm^®^ was treated with phosphate buffered saline (PBS) before being injected with the cells (fluid milieu). There was no significant difference between fluid and dry milieu. Vital cells are shown green (Figure 1). In the PBS pre-treated group cells migrated from a single accumulation at the site of injection on day 1 (Figure 1a) white arrow (red arrow representing basal region) in longitudinal (Day 2, Figure 1b) and transversal (day 3, Figure 1c) direction. This observed pattern of cell migration is the same in the group not pre-treated with PBS (Figure 1d–f). Generally, the migration occurred from the apical (white arrow) to the basal regions (red arrow) (Figure 1h).

**Figure 2:**
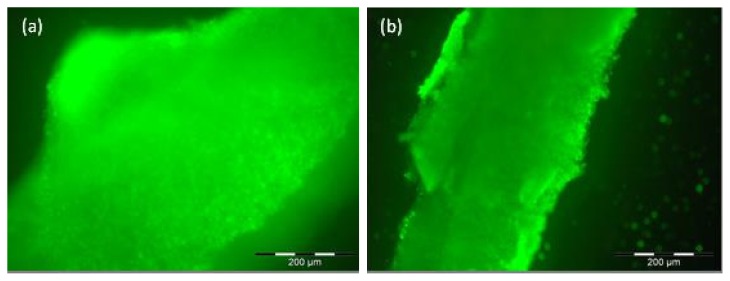
LIVE/DEAD assay: (**a**) Cells of mouse embryonic fibroblasts (MEF) lineage three weeks after injection in Matriderm^®^: Green color represents alive cells through the entire matrix; (**b**) Cells of NIH lineage two weeks after injection in Matriderm^®^: Viable cells are mostly seen on the matrix’ surface but also in deeper layers of the matrix. Scale bar = 200 μm.

Even after two and three weeks, vital cells could be found. Here, the results are shown for the cell lineages MEF (Mouse embryonic fibroblasts) and NIH/3T3 (established from a NIH Swiss mouse embryo) after two and three weeks, respectively (Figure 2).

**Figure 3:**
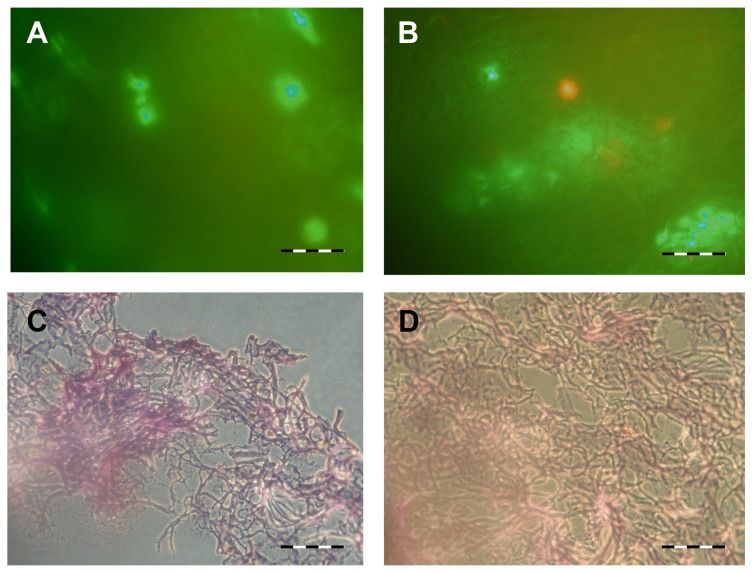
Matriderm was populated by pipetting the cells on the sheets (**A** and **C**) or by centrifuging the cells into the matrix (**B** and **D**). After cultivating for one week in submerged culture, samples were evaluated by LIVE/DEAD staining (**A** and **B**) or H&E staining of the respective cryosection (**C** and **D**). Please note the sparse population of the matrix and the insufficient distribution of vital cells throughout the matrix. Scale bar: A and B = 50 μm, C and D = 100 μm

In order to prove the efficiency of our method, two other protocols were used to incorporate JB6 cells and MEF cells into Matriderm^®^ sheets: we added them on top of the material and centrifuged them into the material under mild centrifugation conditions. LIVE/DEAD analysis was performed one week after seeding. In samples treated with both methods vital cells were observed (Figure 3A,B). Nevertheless, cell density was low compared to microinjected samples. Cells appear rather shapeless and no clear adherence to the material was observed. These results were confirmed by staining cyrosections with hematoxilin and eosin (Figure 3C,D). Vital cells are sparse and insufficiently distributed in the matrix.

### 2.2. Proliferation

**Figure 4:**
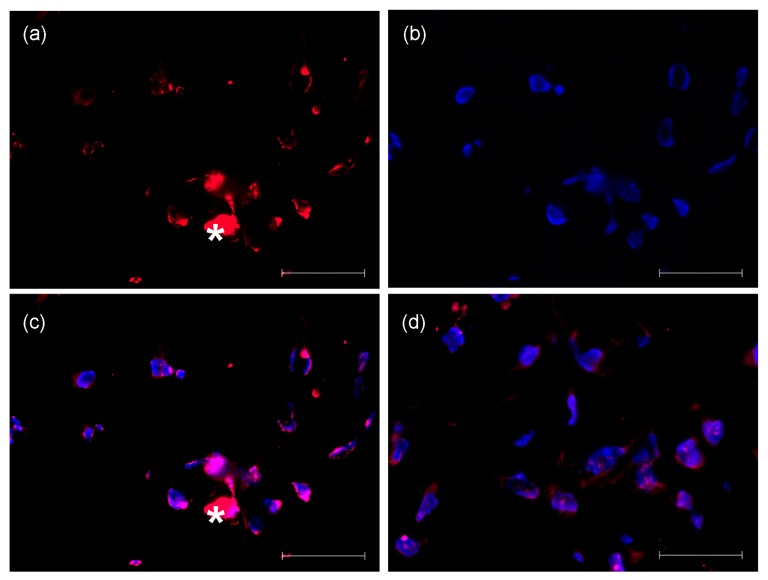
Cdc6 localization suggests proliferation of microinjected JB6 cells in the matrix after three weeks of submerged culture. One millimeter Matriderm^®^ samples injected with JB6 cells were cut into 20 μm sections and stained with cdc6 antibody followed by secondary antibody [purple fluorescence, panel (**a**)]. Nuclei were counterstained with DAPI [blue fluorescence, panel (**b**)]. In panels (**c**) and (**d**), merged images from different sites are shown. Nuclear localization of cdc6 was found in most of the cells, indicating G1 phase, while a proportion of the cells displayed cytoplasmic fluorescence as indicative for S phase [white star, panels (**a**) and (**c**)]. Scale bar = 50 μm.

After three weeks of submerged culture, specimen were fixed and embedded for further analysis. We assessed the presence of proliferating cells using the marker cdc6 which is localized in the nucleus in G1 and in the cytoplasm in S-phase (here demonstrated with JB6 cells, Figure 4) [18]. Purple color represents the cdc6 antigen whereas blue color represents nuclear counterstain. The proliferating cells were distributed evenly throughout the entire matrix without clear preference of the basal regions.

### 2.3. Integration of the Skin Substitute *in Vivo*

**Figure 5:**
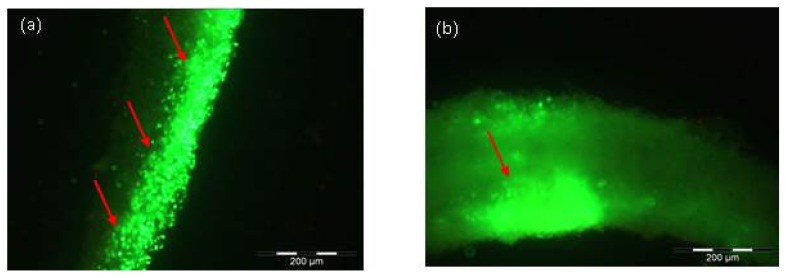
LIVE/DEAD assay one week after transfer of skin substitute into the back of the mouse. (**a**) Vital JB6 cells shown green are predominantly located on the apical part of the matrix (red arrow) but also found in deeper layers; and (**b**) MEF cells show an agglomeration at the site of injection. Both lineages show almost exclusively vital cells (green color). Scale bar = 200 μm.

**Figure 6:**
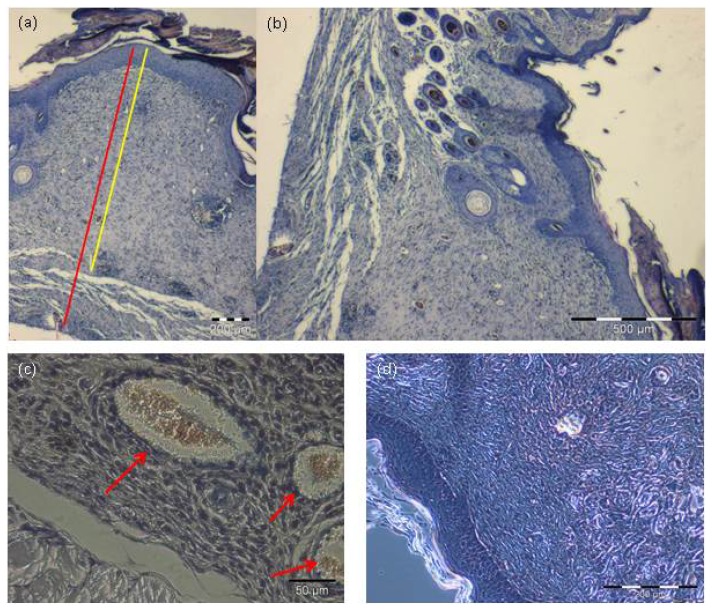
(**a**) The skin substitute presents a complete reepithelialization over a distance of 1.28 mm (yellow line), respectively 1.65 mm (red line); (**b**) The transition to healthy skin is characterized by resemblance of the skin substitute’s and the mouse’s epithelium. The integration of the skin substitute is shown by a newly formed epithelial layer; (**c**) Angiogenesis is seen (red arrows), giving proof of and providing the basis for nourishment of the injected keratinocytes and fibroblasts; and (**d**) Keratinocytes form a distinct layer. (**a**) and (**b**) 4×; (**c**) and (**d**) 10× magnification.

The next step was to investigate if our cell seeded construct integrated into a full thickness wound, if this integration is maintained by a sufficient vascularization and if it is accompanied by epithelialization, in order to prove the feasibility of our approach in the treatment of burn wounds. For *in vivo* analysis, full thickness wounds measuring 1 × 1 cm^2^ were induced on mice’s dorsum. The animals’ dorsum has a reduced likeliness for infections and manipulations by the animal due to its nearly impossible accessibility. After full thickness wound induction, the cell seeded bovine elastin-collagen matrix was transplanted to for permanent wound coverage and sutured into the defect. Directly after surgery, a special wound dressing was performed as explained in detail in the experimental section. No signs of wound infections or matrix rejection could be observed during the three weeks. After three weeks the animals were sacrificed and the tissue removed. The matrix was fully integrated into the skin and the wound showed stable epithelialization after 3 weeks with full wound healing. Seeded Matriderm^®^ sheets showed full integration in the wound model. Viability of cells could be proven in LIVE/DEAD assay (Figure 5). Furthermore, excellent integration concerning epithelial regeneration and stratification could be shown in histological analysis via HE-staining. Thus a fully newly formed keratinocyte layer and angiogenesis could be observed in the matrix. Also, reepithelialization was seen (Figure 6). Altogether, there was no significant difference between the skin substitute and normal skin in the animal. An epithelial stratification resembling healthy skin was observed. The structure of Matriderm^®^ combined with the precise injection technique using micropipettes resulted in an optimized and thorough distribution of the injected cells in longitudinal and in transversal direction. This specific characteristic constitutes the basis for not only a stratified epithelium but for a functional three-dimensional skin substitute. Thus, the epithelium formed by the cultured substitute is hardly different from that of healthy skin.

Previous studies have described the seeding of keratinocytes and fibroblasts on the outer surface of the matrix with a small number of cells only superficially migrating into the matrix [19]. Here, the presence of injected cells throughout the entire thickness of the matrix is provided by a sufficient injection technique which permits the precise seeding of the matrix. This is essential for creation of functional tissue like the subsequent re-epithelialization shown in our data. Besides the viability of the cells, their proliferation could be proven using cdc6 antigen. S phase cells were found regularly among injected JB6 cells without establishing a clear proliferation zone indicative of keratinocyte differentiation. Taking into account the experimental conditions, keratinocyte differentiation was not expected since an air-liquid interface cultivation would have been necessary to induce differentiation.

The formation of a strong keratinocyte layer and angiogenesis indicated functionality of the substitute. Angiogenesis is of eminent importance for the nourishment of the previously seeded cells. In clinical translation this matters in particular in patients with wounds affecting a high TBSA where the central area of the graft often poses a problem in means of tissue nourishment.

Our experiments showed that keratinocytes and fibroblasts that were injected into Matriderm^®^ are able to survive and reassembly within the matrix. Also, the substitute’s successful integration into healthy dermal tissue demonstrated that Matriderm^®^ cultured with epidermal (stem) cells by means of micropipettes is a potential basis for new skin substitutes. Stem cells have known regenerative capacities and can help with graft survival in numerous ways. There have been attempts to use stem cells, *i.e.*, ASCs and human umbilical cord matrix stem cells, in skin flaps showing improved flap survival in animal models [20,21]. In this regard, stem cells pose an important chance in skin equivalents. The combination of the introduced technique of injection and stem cells will be the next step in assumption of an improved functionality of the resulting tissue. In previous studies, Matriderm^®^ has been seeded with pancreatic stem cells showing improved wound healing [22]. Also, the seeding of Matriderm^®^ with preadipocytes has been described, showing difficulties of penetration into deeper layers of the matrix with induced preadipocytes [23]. In the studies describing the seeding of Matriderm^®^ the cells were applied on top of the matrix. Using micropipettes for injection makes it possible to seed cells into the matrix. Our method provides us with prompt availability of a skin graft and permits the seeding of a scaffold with high cell counts making one step further towards off-the-shelf skin substitutes. Matrices such as Matriderm^®^ could be used as a carrier for Epidermal Stem Cells (ESC), hopefully leading to improved regeneration.

Another possible outlook could be injecting genetically modified cells in full-thickness grafts (FTG) in order to achieve immunoprivileged transplants and improve graft take rate. This would especially matter in case of large area transplantation, e.g., in face-transplantation or composite tissue allotransplantation (CTA). For this purpose more long term and *in vivo* studies have to be carried out.

## 3. Experimental Section

### 3.1. Matriderm^®^

Matriderm^®^ is a three-dimensional bovine collagen-elastin matrix consisting of bovine collagen types I, III and V. It is available in thicknesses of 0.5, 1 and 2 mm. The matrix is commercially distributed by medskin solutions Suwelack A.G., Billerbeck, Germany. For this study 1 mm and 2 mm matrices were used.

### 3.2. Cell Culture

The cells used in this study originate from prefabricated lineages. The NIH/3T3- and the MEF-lineage both are murine embryonic fibroblasts obtained by ATCC. The JB6- lineage is keratinocytes derived from murine epidermal tissue. NIH/3T3 cells were cultured in DMEM high glucose (Biochrom^®^), 1% penicillin/streptomycin, 1% sodium-pyruvate and 1% nonessential amino acids (NEA). The cells were incubated at 37 °C with 5% CO_2_. They were passaged after having been washed with phosphate buffered saline (PBS) and detached by 0.05% trypsin EDTA. Passaging was performed at 60%–70% confluency which was attained after two to three days.

### 3.3. Injection in Matriderm^®^

**Figure 7:**
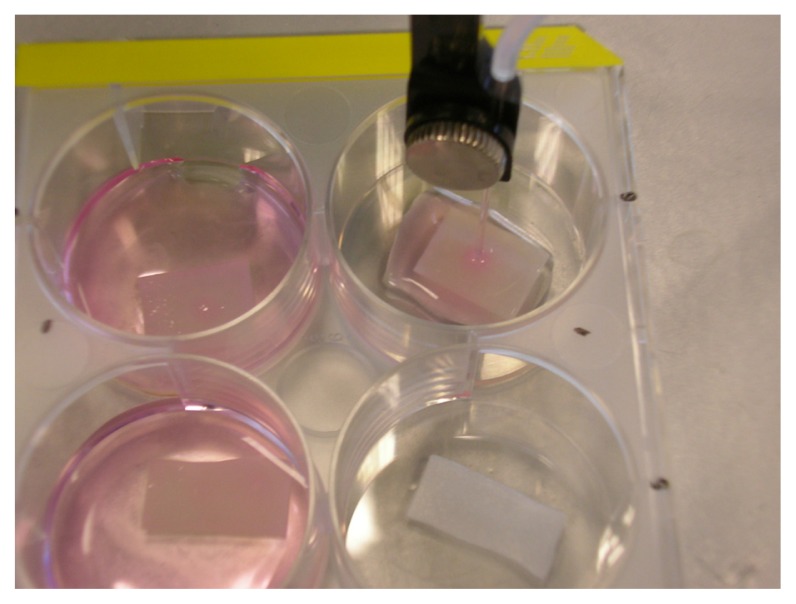
Injection of Matriderm^®^ using stereotactic frame injection, which allows exact cell seeding into the matrix.

**Figure 8:**
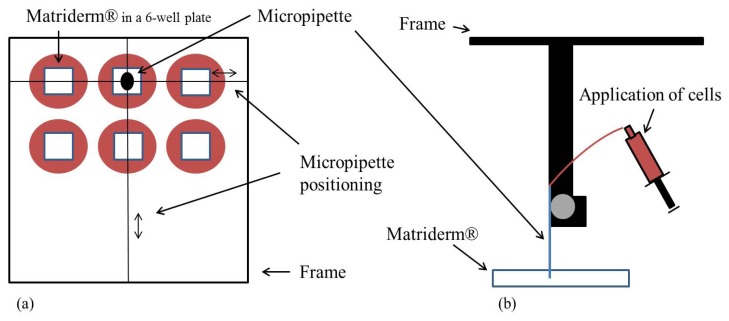
(**a**) Sheets of Matriderm^®^ measuring 1 × 1 cm^2^ are placed in a 6-well plate. Longitudinal bidirectional positioning of the micropipette is possible by means of a frame in which the pipette is mounted; and (**b**) The depth of injection (transversal positioning) is defined via a screw that controls the movement and therefore the location of the micropipette in Matriderm^®^. The cells are applied from a syringe that is connected to the micropipette by a line.

Pointed micropipettes with a 30 μm opening specifically designed for this purpose were used for injection of the cell suspension. A volume of 1 mL was injected containing approximately 3 × 10^5^ cells. The injection was performed using a stereotactic frame allowing us to precisely inoculate the matrix with the epidermal cells and fibroblasts (Figure 7). Therefore a frame was placed around a 6-well plate. In each of the plates a Matriderm^®^ sheet measuring 1 × 1 cm^2^ was placed. Consequently microinjection of the cells was performed. The exact position of the micropipette was achieved using the frame for longitudinal and a screw for transversal positioning. Matriderm^®^ was then centrally injected with the preliminarily cultured cells in a defined depth of the matrix (Figure 8).

### 3.4. LIVE/DEAD Assay

Invitrogen’s Viability/Cytotoxicity Kit LIVE/DEAD^®^ assay was used for assessment of cell viability in the *in vitro* part as well as in the *in vivo* part of the study. It uses calcein AM on the one hand to show intracellular esterase activity resulting in green cytoplasmatic fluorescence representing viable cells. On the other hand dead cells are represented by red fluorescence caused by ethidium homodimer-1 which can only penetrate through defective cell membranes and subsequently interact with nucleic acids. LIVE/DEAD^®^ assay is commercially available.

### 3.5. Immunofluorescence

Cdc6 was described in budding yeast. It is a marker commonly used for proliferation and is essential for DNA replication [24]. We used Molecular Probes’ Anti-cdc6 (human), Mouse IgG_2a_ Monoclonal 37F4 (A-21286). Depending on the cell cycle’s phase cdc6 is either expressed in the nucleus (G1 phase) or in the cytoplasm (S phase).

DAPI (4′,6-diamidino-2-phenylindole) forms a fluorescent complex with A-T rich DNA regions and is therefore used for nuclear staining, here resulting in blue fluorescence [25].

### 3.6. Insertion of the Skin Substitute in Mice

**Figure 9:**
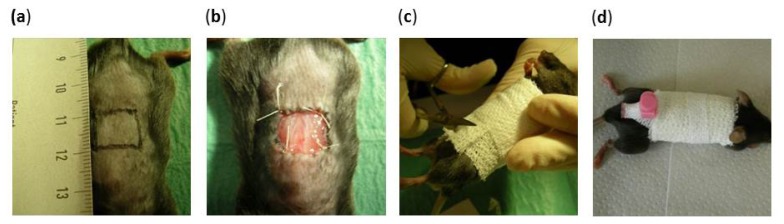
(**a**) 1 cm^2^ of skin on the mouse’s back is excised creating a full-thickness wound; (**b**) Matriderm^®^ treated with epidermal cells is sutured into the defect; (**c**) Sterile wound dressing is applied; and (**d**) An intravenous-needle is placed beneath the dressing for moistening the substitute to prevent drying.

The mice were anaesthetized with 2% isoflurane via mask. An area of 1 cm × 1 cm of full-thickness dorsal skin was excised. Matriderm^®^ treated with the keratinocytes respectively fibroblasts was subsequently sutured under sterile conditions into the just generated skin defect. 6-0 monofil suture material in and fibrin glue were used. The wound was dressed sterile afterwards. In order to keep the wound surface from drying out an intravenous-needle was placed beneath the wound dressing. The wound was flushed with saline intermittently (Figure 9).

### 3.7. Histological Analysis

HE-staining was used for histological analysis. Probes were fixed in 4% buffered formalin for at least five days. Subsequently they were rinsed in water in order to wash out formalin. The following preparation of the probes for embedding was done using an embedding-automat which applies an ascending alcohol line (50%-70%-80%-96%-96%-100%-100%-100%-xylol-xylol-paraffin-paraffin) to the probes. Afterwards they were embedded in 60 °C paraffin and hardened at −20 °C for at least 2 h. The sectioning of the probes was performed by microtome producing 5 μm sections. The subsequent deparaffination used a descending alcohol line (xylol-xylol-100%-96%-80%-70%). The probes were then stained.

### 3.8. Animal and Ethics Statement

All animal experiments were evaluated and approved by the responsible animal care committee (Nds. Landesamt für Verbraucherschutz und Lebensmittelsicherheit) and the Hannover Medical School (Institut für Versuchstierkunde). The experiments were approved under the number 33.12-42502-04-06/1226.

## 4. Conclusions

Our experiments could demonstrate long-term cell survival, migration and proliferation of keratinocytes and fibroblasts up to three weeks after microtransplantation into the porous elastin-collagen matrix as cultured skin equivalents (CSE) *in vitro*. Furthermore, it has been shown *in vivo* that CSE represent a possible treatment for full thickness skin defects. Thus, the establishment of a technique for injecting skin cells was successful. This is a fast and reliable method to create artificial and three-dimensional patterns in the elastin-collagen based matrix serving as a carrier for the cells. The creation of patterns allows for the establishment of functional tissue units depending on the combination of chosen cell types. Our aim is to establish microenvironments for epidermal skin cells which will contribute to a functionalization of tissue-engineered skin. To this end, the application of epidermal stem cells using our method of injection will be the next step. There is hope and partial proof that stem cells further improve wound healing and skin integrity. Further long term and clinical studies have to be carried out.
